# Genomic relatedness and diversity of Swedish native cattle breeds

**DOI:** 10.1186/s12711-019-0496-0

**Published:** 2019-10-02

**Authors:** Maulik Upadhyay, Susanne Eriksson, Sofia Mikko, Erling Strandberg, Hans Stålhammar, Martien A. M. Groenen, Richard P. M. A. Crooijmans, Göran Andersson, Anna M. Johansson

**Affiliations:** 1 Department of Animal Breeding and Genetics, Swedish University of Agricultural Sciences, Uppsala, Sweden; 2 VikingGenetics, Viking Genetics Sweden AB, Skara, Sweden; 3 Animal Breeding and Genomics, Wageningen University & Research, Wageningen, The Netherlands

## Abstract

**Background:**

Native cattle breeds are important genetic resources given their adaptation to the local environment in which they are bred. However, the widespread use of commercial cattle breeds has resulted in a marked reduction in population size of several native cattle breeds worldwide. Therefore, conservation management of native cattle breeds requires urgent attention to avoid their extinction. To this end, we genotyped nine Swedish native cattle breeds with genome-wide 150 K single nucleotide polymorphisms (SNPs) to investigate the level of genetic diversity and relatedness between these breeds.

**Results:**

We used various SNP-based approaches on this dataset to connect the demographic history with the genetic diversity and population structure of these Swedish cattle breeds. Our results suggest that the Väne and Ringamåla breeds originating from southern Sweden have experienced population isolation and have a low genetic diversity, whereas the Fjäll breed has a large founder population and a relatively high genetic diversity. Based on the shared ancestry and the constructed phylogenetic trees, we identified two major clusters in Swedish native cattle. In the first cluster, which includes Swedish mountain cattle breeds, there was little differentiation among the Fjäll, Fjällnära, Swedish Polled, and Bohus Polled breeds. The second cluster consists of breeds from southern Sweden: Väne, Ringamåla and Swedish Red. Interestingly, we also identified sub-structuring in the Fjällnära breed, which indicates different breeding practices on the farms that maintain this breed.

**Conclusions:**

This study represents the first comprehensive genome-wide analysis of the genetic relatedness and diversity in Swedish native cattle breeds. Our results show that different demographic patterns such as genetic isolation and cross-breeding have shaped the genomic diversity of Swedish native cattle breeds and that the Swedish mountain breeds have retained their authentic distinct gene pool without significant contribution from any of the other European cattle breeds that were included in this study.

## Background

Based on the type of livestock management, European cattle breeds can be broadly categorized into either commercial or traditional/native cattle. Commercial cattle breeds are mainly used in intensive animal farming, which aims at maximizing the overall production and economic profit, with a few popular breeds being disseminated throughout the world [1, 2]. In contrast, traditional cattle breeds have a long history of adaptation to their respective environments [3]. Whereas commercial cattle far exceed the traditional cattle breeds in terms of milk and meat production, the latter breeds have great cultural value, are often adapted to the environment and climate conditions in which they are bred, and sometimes display a few superior production or functional traits compared with commercial cattle. For instance, Fjäll cattle produce milk with a superior protein composition that is particularly well-suited for cheese-making compared to commercial cattle such as Holstein-Friesian. In addition, a study, which compared the grazing pattern of Holstein-Friesian and Fjäll cattle in a grass-dominated pasture area, showed that the latter travelled over a larger area and had preferences towards diverse vegetation types [4, 5]. Thus, traditional/native cattle breeds can be valuable for grassland management with diverse vegetation.

In a recent FAO report [6], many cattle breeds were classified “at risk”. It should be noted that this classification was based only on a fraction of all the native breeds that currently exist worldwide. The diversity status of about 50% of all cattle breeds globally, is currently unknown, and therefore proper conservation strategies cannot be designed for these breeds. Advancement in affordable high-throughput genotyping techniques has made it possible to genotype a large number of molecular markers, i.e. single nucleotide polymorphisms (SNPs), at a reasonable cost. Therefore, it is now feasible to infer the population history and diversity status of breeds for which only scant recorded information is available. In fact, many recent studies have used a relatively large number of SNPs to explore the genetic diversity, demographic history, and relatedness between different traditional Eurasian cattle breeds [1, 7–10]. For example, based on 30,000 SNPs, Mastrangelo and colleagues [1] reported the patterns of gene-flow between different Italian native cattle breeds and detected recent inbreeding in several of them.

In Sweden, the old local bovine breeds predominated until the twentieth century, although importation of some cattle from the Netherlands was documented as early as the sixteenth century [11]. For example, approximately 400,000 Fjäll individuals were raised in the north and middle parts of Sweden by the end of the nineteenth century [12]. With more efficient breeding schemes, targeted use of artificial insemination (AI) bulls, and competition from other breeds, the number of individuals from local breeds decreased rapidly. For instance, the Swedish Red Polled breed counted about 30,000 individuals in the 1930’s, but only 20 cows remained in 1979 [13]. During the more recent years, there has been an increasing interest for local breeds as a cultural and genetic resource and measures have been taken to preserve them.

Swedish native cattle breeds display a large phenotypic diversity in terms of conformation- and production-related traits [14, 15]. For example, Fjäll cattle display a white coat color with black or brown spots, whereas most Swedish breeds such as Swedish Red, Swedish Red Polled, and Ringamåla cattle, display a solid/spotted red coat color. Another example is the large fraction of individuals that display polledness in Fjäll, Bohus Polled and Swedish Red Polled cattle breeds (for more detailed information, see Additional file 1: Table S1). In addition, many of these breeds have inhabited these local regions for many generations and may harbor unique gene variants that underlie adaptation to the local climate. Thus, these traditional cattle breeds are important resources for future breeding programs that focus on novel/alternate breeding goals.

While many studies have investigated the patterns of the genetic diversity and structure in traditional European cattle breeds [1, 7, 10], to the best of our knowledge, there are only a few studies [11, 16–19] on Swedish native cattle breeds. Moreover, these studies included only a small number of Swedish cattle breeds and used either mitochondrial or microsatellite markers. In our study, we genotyped about ~ 140,000 SNPs in 143 bovine samples that represent all the nine native cattle breeds from different parts of Sweden. We carried out standard population genetic analyses that use either independent SNPs or haplotypes with the aim to explore the genetic diversity, demography and relatedness among all the native Swedish cattle breeds defined by the Swedish Board of Agriculture.

## Methods

### Sample collection and DNA extraction

**Fig. 1:**
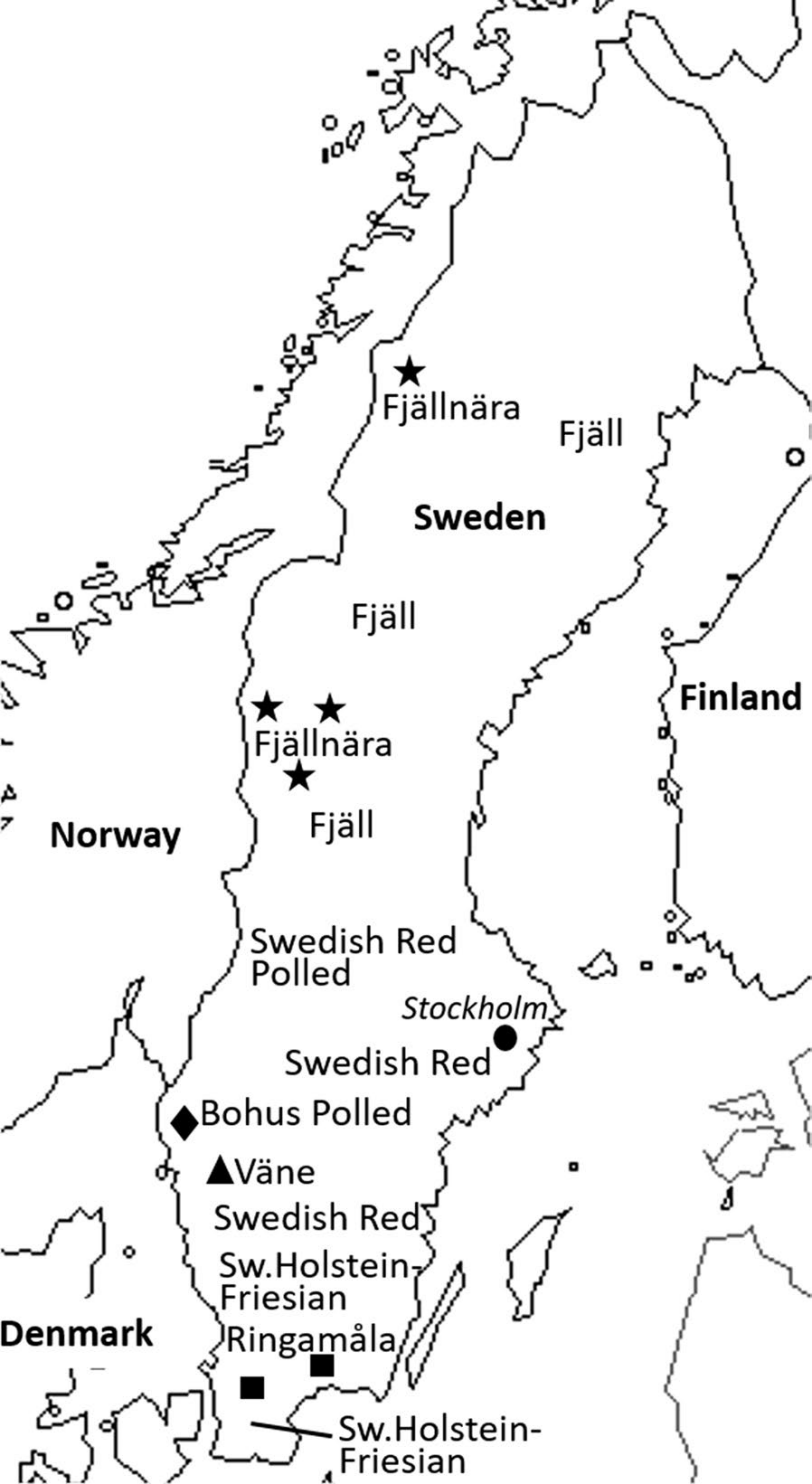
A map of Sweden indicating the approximate geographic origin of the analysed Swedish cattle breeds. Important locations where remaining animals of local breeds were found are marked (star = Fjällnära, diamond = Bohus Polled, triangle = Väne, square = Ringamåla). Some of the breeds originated from a wider geographic area: the Fjäll breed from northern Sweden, the Swedish Red Polled from middle Sweden, the Swedish Red from south middle to southern Sweden, and the Swedish Holstein-Friesian from southern Sweden. The Swedish Polled cattle shares ancestry with the Swedish Red Polled and Fjäll breeds

We genotyped samples from 147 individuals that represented nine Swedish cattle breeds (Fig. 1 and Table 1): 145 samples were obtained from a collection of frozen samples kept in the Department of Animal Breeding and Genetics of the Swedish University of Agricultural Sciences, i.e. old frozen blood samples, semen samples and DNA samples from 35, 51 and 59 individuals, respectively; and nasal swabs were sampled from two additional individuals in 2017. All analysed samples were from animals that were born from the mid-1970s to the early 2000s, except for one Swedish Red polled individual born in 2016. When available, we used information about the farm of origin and pedigree to select animals from various locations and to avoid including very close relatives such as full-sibs or parents and offspring. For DNA extraction from blood samples, we used either a salt-extraction-based method [20] or a QIAsymphony automated platform (Qiagen) following the manufacturer’s instructions. DNA was quantified and quality-controlled using either the Quant-iT™ PicoGreen™ dsDNA Assay Kit (ThermoFisher Scientific, Waltham, MA, USA) or a NanoDrop 8000 Spectrophotometer (ThermoFisher Scientific).

**Table 1:** Genetic diversity summary statistics for the studied Swedish cattle breeds

Breeds	Sample size	P_m_	H_o_	H_e_
Fjäll cattle	23	0.9422	0.3716	0.3578
Bohus Polled cattle	6	0.8187	0.3365	0.3167
Fjällnära cattle	16	0.9253	0.3031	0.3587
Swedish Polled cattle	12	0.9279	0.3836	0.3615
Swedish Red Polled cattle	17	0.9426	0.3622	0.3679
Ringamåla cattle	13	0.8929	0.3350	0.3356
Väne cattle	9	0.7981	0.3348	0.3041
Swedish Holstein-Friesian	24	0.9736	0.3907	0.3883
Swedish Red cattle	23	0.9460	0.3622	0.3606

P_m_: proportion of polymorphic markers, H_o_: average observed heterozygosity, H_e_: average expected heterozygosity

### SNP genotyping and filtering

DNA samples from the 147 individuals were genotyped using the GeneSeek^®^ Genomic Profiler High-Density Bovine 150 K (GGP HD150K) array, with an average SNP spacing of about 19 kb. Genotypes were called using the GenomeStudio^®^ software (Illumina, San Diego, CA, USA) and two samples with a genotyping rate lower than 95% were discarded from the dataset. Furthermore, analysis of the pedigrees revealed that two samples had a foreign origin and were excluded from the dataset. A bash script was used to convert the Illumina genotypes report file to plink-file —“ped” and “map”—format. To ensure that only high-quality SNPs were included in the dataset, we excluded SNPs that were assigned to sex chromosomes and unassembled contigs, SNPs with missing genotypes in more than 5% individuals, and SNPs with a minor allele frequency (MAF) lower than 0.05. All quality-filtering steps were carried out using PLINK 1.9 [21]. Filtering based on Hardy–Weinberg equilibrium was not applied since several of the studied breeds were small and likely sub-structured and influenced by inbreeding and genetic drift. Thus, we expected that there would be deviations from the Hardy–Weinberg proportions.

### Genetic diversity and a recent change in demography

To assess the genetic diversity in each breed, we used the R package *adegenet* [22] to estimate the average observed heterozygosity (H_o_) and the average expected heterozygosity (H_e_). We also estimated the levels of runs of homozygosity (ROH), which are long stretches of identical-by-descent (IBD) homozygous genotypes that can provide valuable insight into recent and past demography of a population [23]. For ROH estimation, we used PLINK (v. 1.9) with default settings except that we allowed only two missing genotypes per window of 50 SNPs (–*homozyg*-*window*-*missing* 2), two heterozygous SNPs along the entire ROH segment (–*homozyg*-*het* 2), and one or more SNPs per 80 kb region (–*homozyg*-*density* 80). In addition, the pattern of linkage disequilibrium (LD) decay between pairwise SNPs was generated for the breeds for which the sample included more than eight individuals to supplement the demographic pattern that was inferred based on ROH. For this purpose, we used SNeP [24] to calculate pairwise r^2^ values between pairs of SNPs located within 2-Mb windows [8, 25].

### Assessment of genetic structure and relatedness among Swedish native cattle breeds

To assess population structure among the nine Swedish native cattle breeds, first we used three approaches that consider individual SNPs: (1) principal component analysis (PCA), (2) admixture analysis, and (3) *F*_st_ and construction of the maximum likelihood-based phylogenetic tree. To perform PCA, the “bed” format of PLINK was converted into “gds” format using the “gdsfmat” package before applying the “*snpgdsPCA*” function of the SNPrelate package [26, 27]. ADMIXTURE [28] was carried out for population cluster analysis, with K-values ranging from 2 to 9. Prior to the ADMIXTURE analysis, LD pruning was performed using the “—*indep”* function in PLINK (v 1.9) to reduce the overall pairwise LD to less than 0.15. The output from ADMIXTURE was visualized using the python package PONG [29]. Pairwise *F*_st_ distance was calculated using the “staMPP” R package [30]. A neighbor-joining (NJ) tree was constructed from the *F*_st_ distance matrix using the “phangorn” R package [31]. We also generated a maximum likelihood (ML) based phylogenetic tree using the “Treemix” software [32].

Finally, the genetic structure/clustering pattern was assessed using the algorithm implemented in CHROMOPAINTER and fineSTRUCTURE [33]. Because these algorithms take phased data as input, we used Beagle 4.1 [34] to phase the genotypes of each chromosome, separately. The underlying algorithm in CHROMOPAINTER considers the pattern of LD and the underlying recombination process along the markers to reconstruct each haplotype of a recipient individual as a series of chunks from the other “donor” individuals. As recommended by [33], first we used the “fs” pipeline to calculate the nuisance parameters: n (similar to effective population size) and M (population mutation rate). The inferred values of these parameters were fixed in CHROMOPAINTER to obtain the ChromoPainter coancestry matrix (count matrix as well as length matrix) that measures the shared haplotypes between samples across the genome. Using the count matrix (number of chunks), which represents the number of haplotypic chunks copied among all individuals, we ran a fineSTRUCTURE analysis to cluster the samples into genetically homogeneous groups. Note that we ran this entire analysis in parallel using fineSTRUCTURE v4 (version 4).

### Genetic relationships of the Swedish cattle breeds with other European cattle

To investigate the relationships between the Swedish native cattle breeds and other European cattle breeds, we recovered bovine SNP genotyping array data for various European cattle breeds previously reported [10, 35–38] from the web-interfaced genetic Diversity Exploration (WIDDE) database [39], and merged these data with the TOP alleles using PLINK (v. 1.9) [21]. In addition, we included genotyping data for an indicine cattle breed, i.e. Gir, because it was used as an outgroup in the phylogenetic analysis. To have a sample size per breed similar to that of the Swedish cattle breeds genotyped in our study, we retained only 24 samples per breed in the analysis. In a later step, we merged these samples with the Swedish cattle samples into a combined dataset that was filtered by applying the following parameters in PLINK (v. 1.9): –*mind* 0.9, –*geno* 0.05, –*maf* 0.05. Sample information for the final merged dataset that was used for the downstream analysis is in Additional file 1: Table S2. We carried out the PCA, admixture, and phylogenetic analyses by using exactly the same pipeline as described previously in this “Methods” section.

## Results

### Genetic diversity indices and demographic inferences

After applying the filtering criteria, the final dataset for the Swedish native breeds consisted of 143 samples and 111,914 SNPs. The genetic diversity summary statistics are in Table 1. A majority of the Swedish native cattle breeds displayed a high proportion of polymorphic loci (P_m_), ranging from 0.7981 in Väne to 0.9736 in Swedish Holstein-Friesian. This indicates that the SNPs selected for the GGD HD150K array are highly informative for Swedish cattle breeds. Similarly, the expected heterozygosity (H_e_) is relatively high with values ranging from 0.3041 in the Väne breed to 0.3883 in the Swedish Holstein-Friesian breed.

**Fig. 2:**
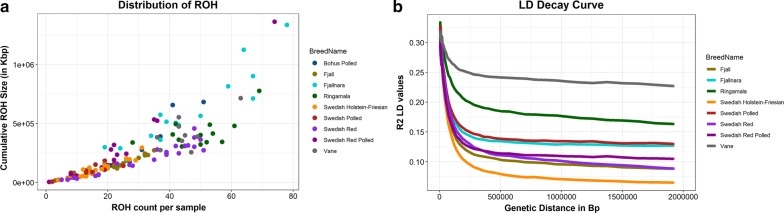
**a** ROH profile of Swedish cattle breeds, where each circle represents an individual and **b** linkage disequilibrium decay in four Swedish cattle breeds for which the sample size was larger than 8

Demographic inferences in Swedish cattle breeds were made based on the analyses of ROH and LD decay. The ROH profile (ROH count and cumulative ROH length) varied a lot both between and within the Swedish cattle breeds (Fig. 2a and see Additional file 1: Table S3). In Fjällnära, the ROH count and cumulative ROH length ranged from 8 to 78, and from ~ 6 Mb to ~ 1.3 Gb, respectively. In Swedish Holstein-Friesian, ROH count ranged from 2 to 31, and cumulative ROH length ranged from ~ 6 Mb to ~ 295 Mb, while the corresponding numbers in Fjäll were 1 to 45, and ~ 4.5 Mb to ~ 310 Mb, respectively. These results indicate relatively large/diverse ancestral populations for Swedish Holstein-Friesian and Fjäll breeds. Swedish Red Polled individuals also displayed a large variation in ROH profile (ROH count ranged from 1 to 74, and cumulative ROH length ranged from ~ 4.3 Mb to ~ 1.3 Gb). Moreover, the cumulative ROH size of some Swedish Red Polled individuals (such as Individual SRP5 and SRP 8) was similar to that of other individuals (such as VAC7 and RMC13), but with lower ROH counts (see Additional file 1: Table S3), which indicates that some mating has occurred between closely related individuals. Conversely, Väne and Ringamåla cattle display relatively large ROH counts and cumulative ROH size, which indicates genetic isolation and a relatively small founder population. As expected, inferences that are drawn based on the pattern of pairwise LD decay (Fig. 2b) are consistent with those from ROH profiles. For example, for the Ringamåla and Väne breeds, we inferred a low level of haplotype diversity since they have the highest r^2^ values at all pairwise SNP distances and an overall slow LD decay. For the Fjäll, Swedish Holstein-Friesian and Swedish Red breeds, we inferred a wide haplotype diversity since they have the lowest r^2^ value at all pairwise SNP distances and a rapid LD decay.

### Genetic structure and relationships among Swedish native cattle breeds

**Fig. 3:**
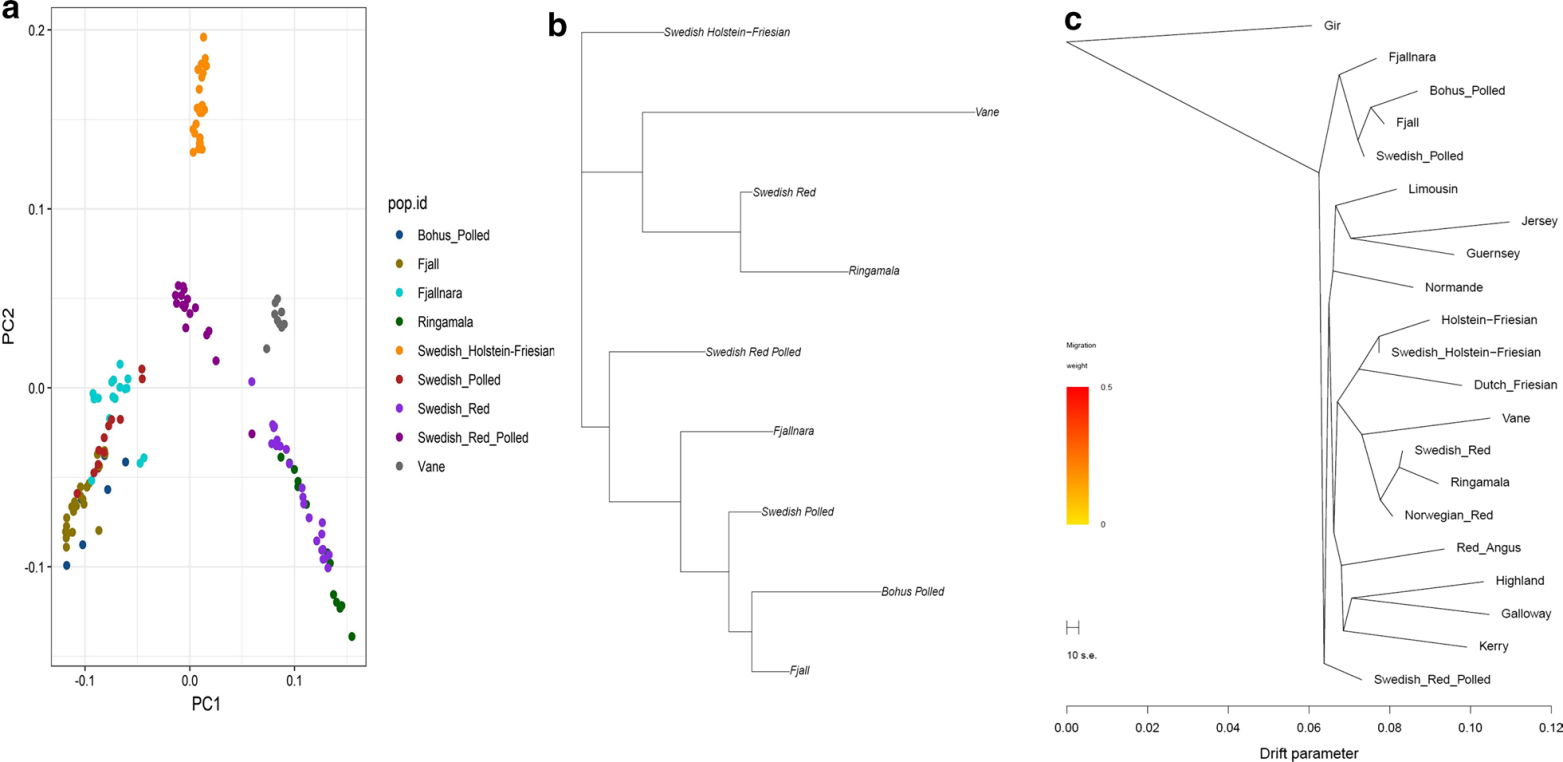
Genetic relatedness among the Swedish cattle breeds using: **a** principal component analysis, **b** F_st_-based phylogenetic tree, and **c** maximum likelihood based phylogenetic tree

To assess the genetic relationships among the Swedish native cattle breeds, first we performed a PCA. The first principal component (EV1), which explains 5.76% of the total variance, clearly separated the Fjäll, Swedish Polled, Fjällnära and Bohus Polled breeds from the Swedish Red, Väne and Ringamåla breeds. The second principal component (EV2), which explains 3.03% of the total variance, separated the Swedish Holstein-Friesian breed from the cluster of native Swedish cattle breeds. Furthermore, among the native Swedish cattle breeds, Swedish Red Polled (which occupies the central position in the plot) and Väne clearly form separate clusters (Fig. 3a), whereas the Fjällnära samples are dispersed across the plot, which indicates either sub-structure or large variability within the population. Moreover, the genetic differentiation between Fjäll, Swedish Polled and Bohus Polled cattle, and that between Ringamåla and Swedish Red cattle are low.

The NJ-tree constructed based on *F*_st_ (Fig. 3b and see Additional file 2: Figure S1), and the ML-based phylogenetic tree (Fig. 3c) are in agreement with the patterns identified by using PCA (Fig. 3a). In general, these phylogenetic trees divide the Swedish native cattle breeds into two clusters: one cluster that comprises the Swedish Mountain cattle breeds, i.e. Fjäll, Swedish Polled, Fjällnära and Bohus Polled, and one cluster that includes the southern Swedish cattle breeds with horns—Swedish Red, Väne and Ringamåla. In particular, these trees also suggest close relationships between the Bohus Polled and Fjäll breeds, and between the Ringamåla and Swedish Red breeds. Moreover, the long branch observed for Väne cattle indicates considerable genetic drift/divergence. Interestingly, similar to the PCA pattern, the Swedish Red Polled breed displays an ambiguous clustering pattern, i.e. in the ML-based phylogenetic tree (Fig. 3c), it does not cluster with any of the Swedish cattle breeds, whereas in the *F*_st_-based phylogenetic tree, it forms a sister clade with the cluster of Swedish mountain cattle breeds.

**Fig. 4:**
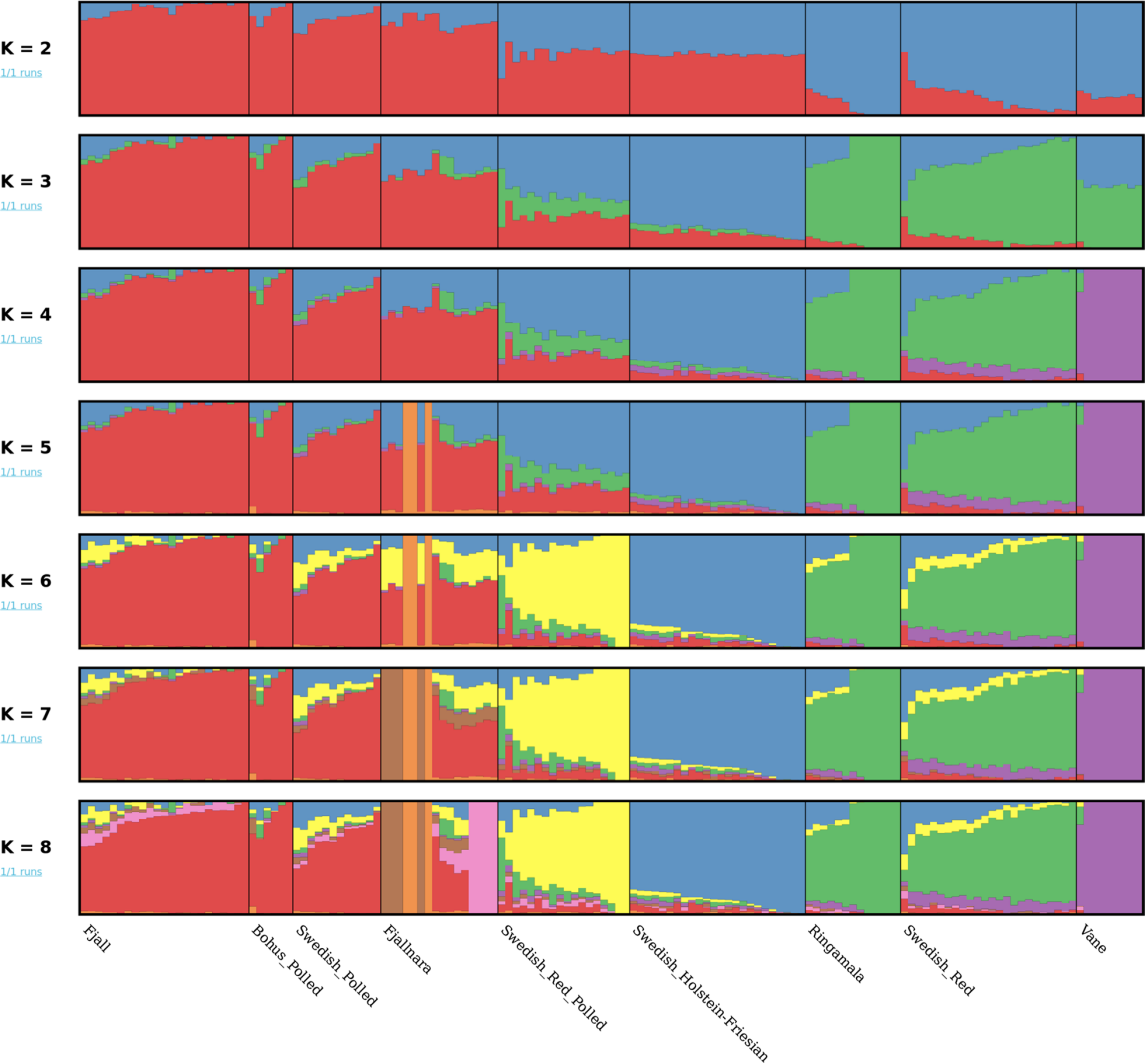
Model-based clustering of Swedish cattle breeds based on the estimated membership fraction of individuals

The clustering pattern (Fig. 4) based on shared ancestry that was inferred from the ADMIXTURE analysis also indicates a first split (at K = 2) between the Swedish mountain breeds (Fjäll, Swedish Polled, Fjällnära, and Bohus Polled) and the southern Swedish breeds (Swedish Red, Väne and Ringamåla). Conditioning the ancestral population to K = 3 separate the Swedish Holstein-Friesian breed from the clusters of native Swedish cattle breeds, and at K = 4, the Väne breed form a separate cluster. Starting from a K value of 5, we observe sub-structures within the Fjällnära breed. Furthermore, at all K values, the Ringamåla and Swedish Red breeds share a remarkably similar distribution of genetic variation. Likewise, Fjäll, Swedish Polled and Bohus Polled cattle also show a similar distribution of genetic variation, which indicates ancestral relatedness. It is worth noting that Swedish Red Polled cattle display a mosaic of ancestries between K = 2 to 5 but forms a separate cluster at K = 6.

**Fig. 5:**
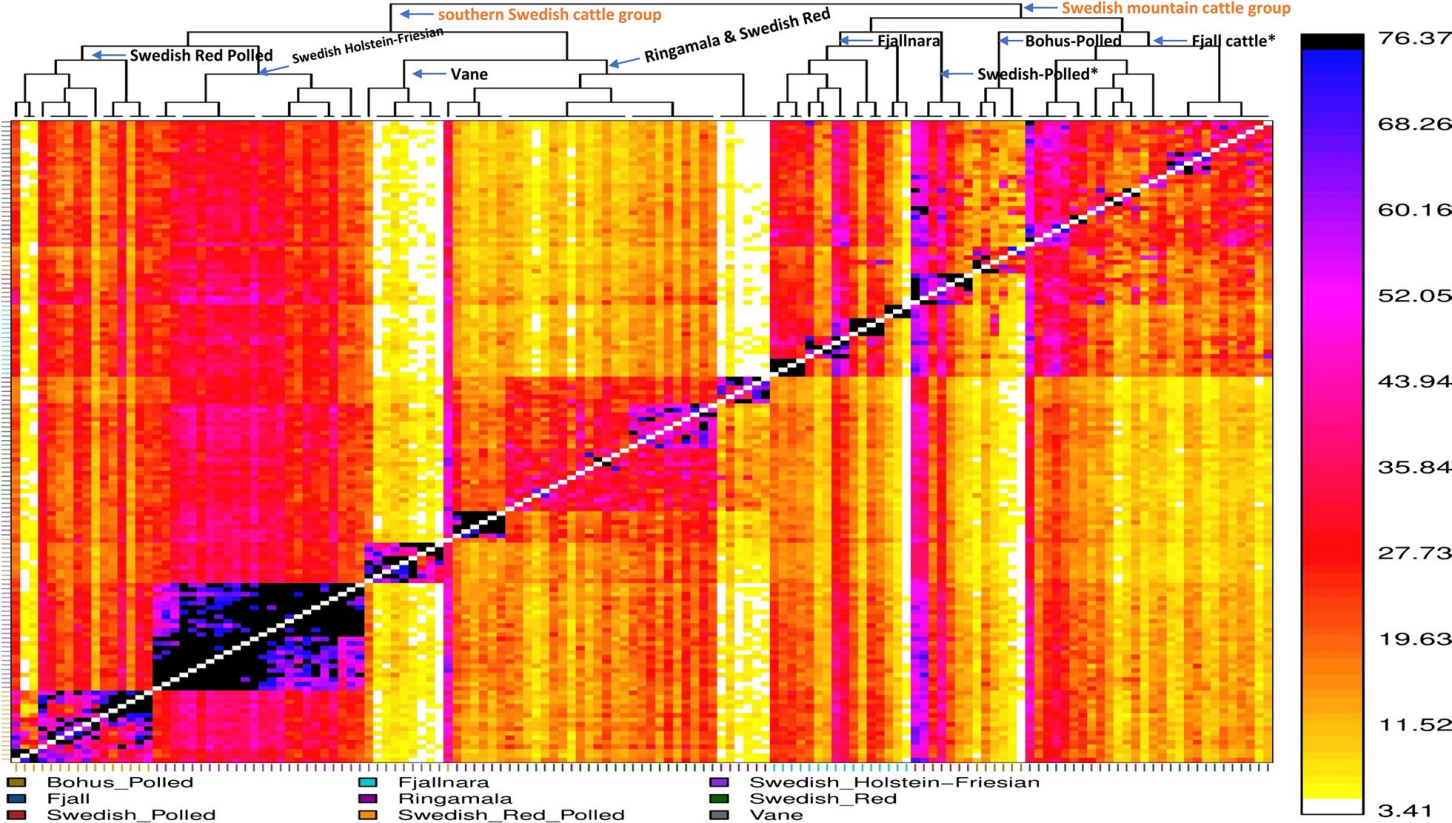
fineSTRUCTURE-based phylogenetic tree. The intensity of colours indicates the number of shared haplotypic chunks. The asterisk (*) indicates low differentiation between Swedish Polled cattle and Fjäll cattle

Haplotype sharing analysis by using ChromoPainter and fineStructure reveals a pattern (Fig. 5) that not only reinforces the findings of all our previous analyses but also refines the clustering pattern of the Swedish native cattle breeds. In particular, these results helped to characterize in more detail the low differentiation between the Swedish mountain cattle breeds. In particular, we observed a low differentiation between Fjäll and Swedish Polled cattle. Interestingly, refined investigation of the Fjällnära individuals revealed four sub-clusters, which correspond to the farms from which the sampled individuals had their ancestries. Furthermore, similar to our previous analyses, the Ringamåla, Swedish Red, and Väne breeds are loosely included in the same cluster. However, Ringamåla and Swedish Red seem to be genetically closer to each other than to Väne. The Swedish Red Polled and Swedish Holstein-Friesian breeds seem to cluster together, but based on the *F*_st_ and PCA analyses, we hypothesize that this is due to the clustering of two other groups (Swedish mountain and southern Swedish breeds).

### Genetic relationships between Swedish cattle breeds and other European cattle breeds

**Fig. 6:**
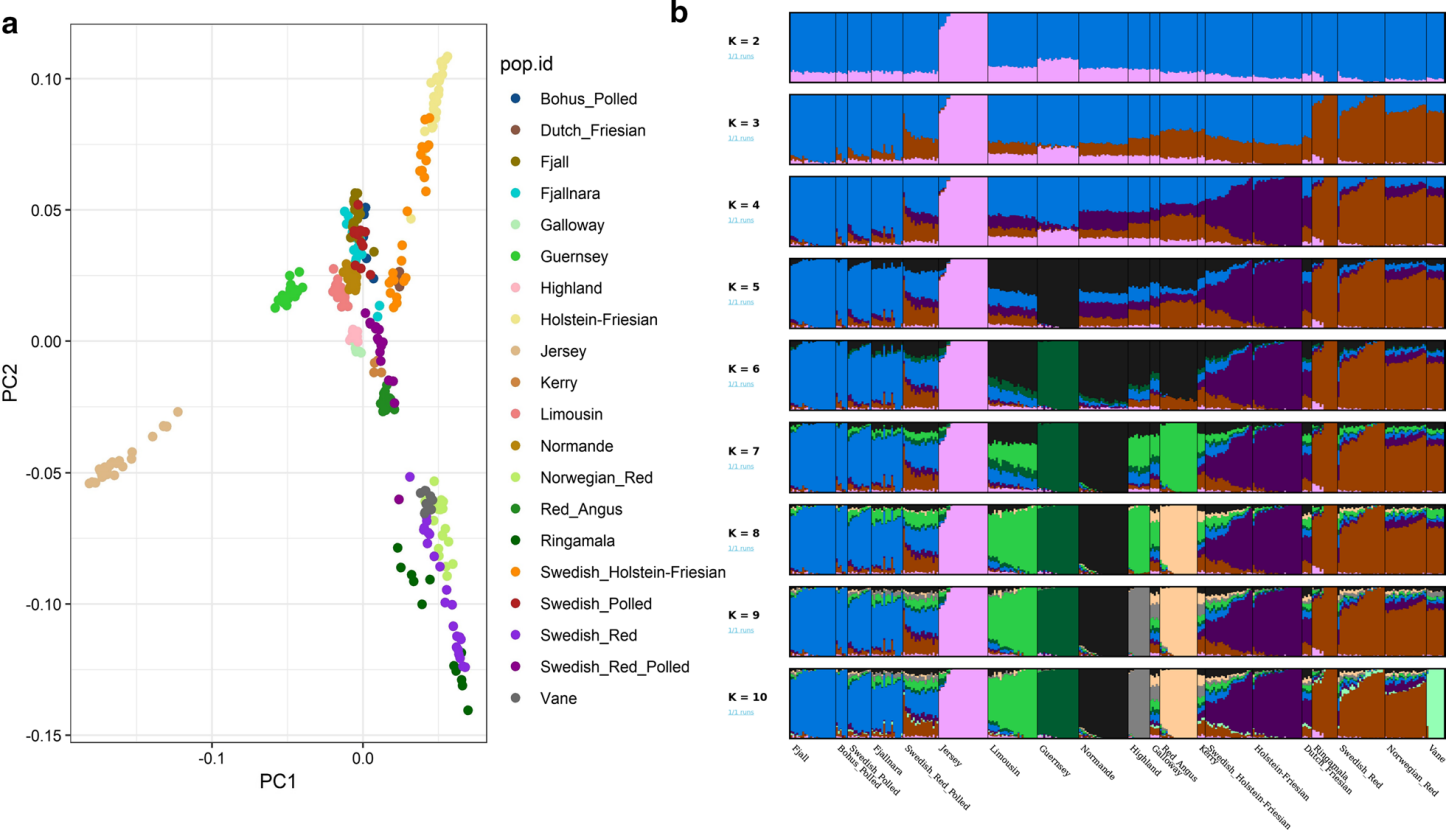
Genetic relationships between the Swedish cattle breeds and several European cattle breeds using: **a** principal component analysis and **b** model-based clustering of different European cattle breeds based on the estimated membership fraction of individuals

We investigated the relationships between Swedish cattle breeds and other European cattle breeds through analyses based on PCA, ADMIXTURE, and phylogenetic trees. The other European cattle breeds mainly originated from north-western Europe, and Norway (see Additional file 1: Table S2). In PCA, the first principal component, which explains 6.25% of the total variance, separated the Jersey breed from all the others (Fig. 6a). This can be attributed to the fact that the Jersey breed has remained genetically isolated for many generations. The second principal component (PC2) separated “red-coloured” cattle breeds (Swedish Red, Ringamåla, Väne and Norwegian Red) from the remaining breeds. As expected, the Swedish Holstein-Friesian individuals cluster with the Dutch cattle breeds. The clustering of “red-coloured” cattle breeds and of the Holstein-Friesian-derived breeds is also observed in the phylogenetic trees (Figs. 3c and 6b). Interestingly, we did not identify any historical relatedness between the Swedish mountain cattle breeds and the European cattle breeds studied here, which probably means that their contribution was not significant.

## Discussion

Rare native cattle breeds are vulnerable and represent important genetic resources since they harbour unique gene pools that result from long-term adaptation to the local environment. Because most of these breeds have not been under intense selection for production traits, they may carry more diverse alleles than their commercial counterparts. For example, based on a high-density genome-wide SNP analysis, Herrero-Medrano et al. [40] identified about 100 non-synonymous polymorphisms that were nearly fixed in commercial breeds whereas, at these same positions, a relatively high frequency of alternative alleles was found in local cattle breeds. Therefore, conservation of local cattle breeds is necessary to preserve these diverse gene pools. However, the development of optimal conservation strategies requires sufficient knowledge about the genetic diversity and population structure of a breed. To achieve this objective in the case of native Swedish cattle breeds, we genotyped nine Swedish cattle breeds with about 140,000 SNPs and analyzed the data using standard techniques of population genetics. Our results suggest varying degrees of genetic diversity and historical relatedness among the Swedish cattle breeds.

### Genetic diversity and demography

The relatively high average expected heterozygosity and proportion of polymorphic SNPs among the Swedish cattle breeds (Table 1) indicate that the SNPs selected on the GGP HD150K array are polymorphic in these breeds. SNPs on this same 150 K array were also found to be highly polymorphic in Russian cattle breeds [8]. However, the possibility of ascertainment bias cannot be ruled out since we observed the largest proportion of polymorphic markers in Swedish Holstein-Friesian, which has a history of recent cross-breeding with Dutch dairy cattle breeds. The overall summary statistics (P_m_ and H_e_) indicate low genetic diversity in the Väne, Bohus Polled and Ringamåla breeds. Inferences drawn based on ROH pattern and LD-decay (Fig. 2) provide further insight into the demographic changes that led to the low level of genetic diversity in these breeds. Ringamåla and Väne cattle display an overall large ROH count and cumulative length as well as the highest r^2^ values across the entire pairwise distances up to 2 Mb. These patterns indicate high autozygosity in the genome of Ringamåla and Väne individuals, which can be attributed to small founder populations that result in ancestral relatedness and lack of gene flow from distantly related populations due to isolation. These inferences are consistent with the known recorded history for these breeds (see Additional file 1: Table S1), which have been maintained isolated from the other Swedish dairy breeds for a long time and have very small-sized populations (see Additional file 1: Table S1) [14, 15]. Interestingly, the relationship between ROH count and cumulative length (Fig. 2a) in the Ringamåla and Väne breeds is similar to the trend observed for other breeds, which indicates a low frequency of long ROH. Long ROH are often the consequence of consanguineous mating resulting in a low-level recombination since there has not been enough time to break the haplotypes. Therefore, a low frequency of long ROH is an indication that the breeding management of Ringamåla and Väne cattle succeeded in avoiding mating between closely-related individuals.

The overall summary statistics, i.e. P_m_, and H_e_ (Table 1) indicate a relatively high genetic diversity in the Fjäll and Swedish Red breeds. Furthermore, these breeds also display relatively small ROH counts and cumulative lengths as well as rapid LD-decay (Fig. 2), which is an indication of high haplotype diversity in these populations. Before the beginning of the twentieth century, Fjäll cattle had a large population size and were phenotypically diverse [14, 15], but thereafter the effective population size of the breed declined until the end of the twentieth century because as intensive farming developed, other breeds were preferred. Our results suggest that in spite of this reduction in population size, the genetic diversity of the Fjäll breed is still significant, probably as a result of using distantly related purebred individuals in breeding programs. The high genetic diversity in the Swedish Red breed can be attributed to the fact that its gene pool has been influenced by the introduction of other Nordic red cattle breeds. Indeed, our analyses (Fig. 6a, b) revealed that the Swedish Red and Norwegian Red breeds cluster together. The large variation in ROH profile found for the Fjällnära breed may be related to sub-structuring in the population that we identified using ADMIXTURE and fineStructure analyses (Figs. 4 and 5). Moreover, unlike for the Fjäll breed, several Fjällnära individuals display an abundance of ROH counts and a large portion of their genome in ROH, which indicate that mating between closely-related individuals occurred rather frequently.

### Genetic structure of the Swedish native cattle breeds

The clustering pattern of the Swedish cattle breeds (Figs. 3, 4 and 5) is in concordance with their known history (see Additional file 1: Table S1). Based on their shared ancestry, we identified two major clusters within the Swedish cattle breeds studied here. Low differentiation between the Fjäll, Bohus Polled, and Swedish Polled breeds in the first cluster was observed in all the analyses (Figs. 3, 4 and 5). Indeed, the Swedish Polled breed was formed in 1938 by merging Swedish Red Polled and Fjäll cattle [15]. Although the two original breeds were crossbred several times, purebred groups of each breed were also maintained. Swedish Red Polled and Fjäll cattle became separate breeds again in 1984 and in 1995, respectively; however, Swedish Polled cattle persists as a separate breed. Attempts have been made to improve the production of Swedish Polled cattle by crossbreeding with commercial cattle breeds. Bohus Polled cattle originates from the south-western part of Sweden (Fig. 1) and it is phenotypically similar to Fjäll cattle. Moreover, the use of semen from Fjäll bulls in Bohus Polled cattle has also been documented. Earlier studies [17, 18] based on analyses with microsatellite markers in northern European cattle, also assigned the Bohus Polled breed to the cluster that includes the Fjäll and Fjällnära breeds. Similarly, the Fjällnära breed was recognized as a subpopulation of Fjäll cattle that has not been intensively selected for milk production. Interestingly, we also identified sub-structures within the Fjällnära population using ADMIXTURE and fineStructure based analyses (Figs. 4 and 5), which correspond to the farms from which these sampled individuals have their ancestry.

The second cluster (Figs. 3, 4 and 5) includes the Ringamåla, Swedish Red and Väne breeds. The Ringamåla breed, which originates from southern Sweden (Fig. 1) shares some resemblance with the Swedish Red breed, and it has been suggested that these two breeds share some ancestry [15]. In addition, the clustering pattern (Fig. 5) inferred from the fineStructure-based analysis indicated a low differentiation between the Swedish Red and Ringamåla breeds. Based on ADMIXTURE and fineStructure analyses, we also observed two sub-structures in the Ringamåla population, which suggests that the origin of the samples studied could come from different farms. Based on PCA (Fig. 3) and ADMIXTURE analyses (Fig. 4), we conclude that Väne is the most diverged breed in this cluster, possibly as a result of drift due to genetic isolation. Swedish Red Polled occupies an intermediate/central position on the PCA plot (Fig. 3a), and shows an ambiguous clustering pattern (Figs. 3b, c), and some within-breed sub-structuring based on the fineStructure analysis (Fig. 5).

Genetic comparison of Swedish cattle breeds with other European cattle breeds revealed that native southern Swedish breeds—particularly Swedish Red and Ringamåla cattle— seem to have been influenced by Nordic red cattle breeds (Fig. 6a and see Additional file 2: Figure S1). Similarly, our analyses also confirmed the historical relatedness between Swedish Holstein-Friesian and Dutch cattle breeds. Interestingly, none of the Swedish mountain cattle breeds displayed genetic proximity to any of the European cattle breeds included in our comparison, which indicates that non-indigenous cattle breeds have had little genetic influence on the Swedish mountain cattle breeds. However, it is necessary to genotype a much larger number of individuals from Norwegian or Finnish cattle breeds to investigate comprehensively the genetic relatedness between different Nordic cattle breeds.

## Conclusions

To conclude, we provide the first detailed analysis of the genetic relatedness and diversity of all Swedish native cattle breeds under the auspice of the Swedish Board of Agriculture. Our findings will aid in the conservation management of these breeds and demonstrate that the magnitude of the genetic drift in some Swedish cattle breeds (such as Väne and Ringamåla) is relatively large, and thus, they require special attention for conservation. Moreover, we also show that the Swedish Mountain cattle breeds (including Fjällnära) are unique in that they have maintained authentic local ancestry. Future studies should aim at genotyping a larger number of individuals using high-density genome-wide SNP arrays or whole-genome sequencing approaches, to help identify the genetic factors involved in their adaptive potential.

## Supplementary information

**Additional file 1: Table S1.** Overview of the Swedish cattle breeds. **Table S2.** Information on the samples included from the previous studies. **Table S3.** Cumulative ROH count, ROH length (kb), and average ROH size (kb) per individual.

**Additional file 2: Figure S1.** *F*_st_-based phylogenetic tree showing the relationships between different European cattle breeds. The following abbreviations are used: JER-Jersey, GNS: Guernsey, LMS: Limousin, NOR: Normande, KC: Kerry cattle, RAN: Red Angus, GA: Galloway, HL: Scottish Highland cattle, DF: Dutch Friesian, SHF: Swedish Holstein-Friesian, HOL: Holstein-Friesian, VAC: Väne cattle, NRC: Norwegian Red cattle, SRC: Swedish Red cattle, RMC: Ringamåla cattle, SRP: Swedish Red Polled, FNC: Fjällnära cattle, SPC: Swedish Polled cattle, SMC: Swedish Mountain cattle (Fjäll cattle), BHP: Bohus Polled cattle.

## Data Availability

The genotyping data was deposited into the DRYAD public data repository upon acceptance of the manuscript. https://datadryad.org/stash/dataset/doi:10.5061/dryad.wdbrv15j4
