# Supplementary material for: Genomic relatedness and diversity of Swedish native cattle breeds
Source: Genet Sel Evol. 2019 Oct 2;51:56. doi: 10.1186/s12711-019-0496-0 (PMC6775670; doi:10.1186/s12711-019-0496-0)
Supplement: Supplementary file 1 — Additional file 1: Table S1. Overview of the Swedish cattle breeds. Table S2. Information on the samples included from the previous studies. Table S3. Cumulative ROH count, ROH length (kb), and average ROH size (kb) per individual. [file 12711_2019_496_MOESM1_ESM.docx]

**Table S1 Overview of the Swedish cattle breeds**

| Breed | Swedish name of breed# | Geographic origin | Genetic evaluation | No. of animals* | Horn/ polled | Colour | Current use and characteristics | Background and year of origin** |
| --- | --- | --- | --- | --- | --- | --- | --- | --- |
| Fjäll | Fjällko | Northern Sweden | yes | 2600 | Polled | Mostly white; white speckled with black or red; colour-sided black or red; more rarely solid red, black or grey | Dairy breed, often used for production of local cheese due to favourable milk protein composition (high frequency of Kappa Kasein B). Small body size of cows ~400-450 kg, and said to be hardy and hold strong ability to find food in natural pastures. | Breed standard and herdbooks established 1893. The breed is believed to orginate from cattle of eastern origin kept in northern Sweden, Finland and Norway since over 1000 years. About 400,000 cows of local breeds were present in northern Sweden by the end of the 19^th^ century. |
| Fjällnära or Unique Swedish mountain cattle | Fjällnära ko | Northern Sweden | No | 300 | Polled | As Swedish Mountain cattle, but with a larger proportion of non-white animals. | On average somewhat smaller than the Swedish Mountain cattle, and with lower milk production. Used for milk production or as suckler cows. | Recognised as a subgroup of Swedish mountain cattle in 1980ies. Originates from few, rather isolated, mountain farms with focus on subsistence farming. |
| Swedish Polled | Svensk kullig boskap (SKB) | Northern and middle Sweden | Yes | 632 | Polled | Mainly as Swedish Mountain cattle or Swedish Red polled. | See Swedish Mountain cattle and Swedish Red polled, but somewhat stronger focus on milk production. | This breed was founded by merging herdbooks for Swedish Mountain cattle and Swedish Red polled in 1938, but the crossbreeding was limited. Some crossbreeding with commercial cattle breeds was also practised. |
| Swedish Red polled | Rödkulla | Middle part of Sweden + imports from Norway and Finland | Yes | 1600 | Polled | Most commonly solid red in different shades, but white markings and black shades occur. | Originally a dairy breed, but today the focus is on conservation of the breed. Mainly kept as suckler cows for beef production and to keep an open landscape. Said to be hardy. Live weight of cows 350-600 kg. | Breed association formed 1912. In the late 1930ies there were about 30,000 cows. The breed went through a bottleneck in the 1970ies, when only ~20 cows remained. Import of polled red animals from western Finland in the 1940-50ies, and from Norway in the 1980ies to reduce inbreeding. |
| Bohus Polled | Bohus-kulla | Swedish west coast, close to Norway | No | 117 | Polled | Similar to Swedish Mountain cattle | Originally a dairy breed, today kept for milk or beef production. Said to be hardy and healthy. Live weight of cows ~400 kg. | Discovered 1993. The current population originates from few herds, that used local bulls, but also some inseminations with Swedish Mountain cattle bulls. |
| Väne | Väneko | South-western Sweden | No | 235 | Horn (two different types of horn shapes) | Different colours occur, often white-speckled or colour sided, but also solid colours | Used for subsistence farming, or as suckler cows on natural pastures. Said to be hardy and with strong ability to find food. Live weight of cows >500 | Discovered 1992 in an isolated herd. |
| Ringamåla | Ringamåla-ko | Southern Sweden | No | 254 | Horn | Red with white markings | Similar usage as Väneko. Live weight of cows 400-500kg | Discovered 1994. Originates from few herds that did not use AI, believed to have a common origin with the commercial Swedish Red breed. |
| Swedish Red | Svensk Röd och vit Boskap (SRB) | Southern and middle Sweden and imported Ayrshire, also imports of other breeds | Yes, genomic (joint Nordic) | 76 628 | Horn | Red with white markings | A commercial dairy breed. Live weight of cows 550-650 kg. | Originates from older type of red cattle from southern and middle Sweden, improved by crosses with Shorthorn and Ayrshire. Official breed formation in 1928, when merged with Ayrshire. Influence from other Nordic red cattle breeds, and to a smaller degree of Brown Swiss. |
| Swedish Holstein- Friesian | Svensk Låglands-boskap (SLB) (in recent years Svensk Holstein) | Imports from the Netherlands and Germany, recently imports of American Holstein | Yes, genomic (joint Nordic) | 125 080 | Horn (polled exist) | Black and white patched | The old Friesian type was more of a dual-purpose breed. Today the breed is a  commercial dairy breed. Live weight of cows ~700 kg. | The Swedish Friesian originates from Dutch and German animals imported in the late 19^th^ century. Herd book was established in 1891, and breed association in 1913. As a result of increasing use of U.S. Holstein bulls, the Swedish Friesian gradually changed into Swedish Holstein from the 1970ies until the mid 1990ies. Few animals of the original Friesian type remains. |

*Number of Swedish red, Swedish Holstein-Friesian, Swedish Polled in 2018 according to Växa Sverige Cattle statistics 2019 (https://www.vxa.se/globalassets/dokument/statistik/husdjursstatistik-2019.pdf). Number of Väneko, Ringamålako and Bohus Polled cattle in Januay 2018 according to Föreningen Allmogekon (www.allmogekon.se). Number of Swedish Mountain cattle and Fjällnära cattle in 2016 according to Svensk Fjällrasavel ([www.fjallko.se](http://www.fjallko.se)), with the number of Fjällnära included in the number of Swedish mountain cattle. The association Föreningen för äldre svensk boskap ([www.boskap.nu](http://www.boskap.nu)) recognises a lower number of Fjällnära as pure Fjällnära. Number of Swedish Red Polled in 2014 according to Svensk rödkulleförening (www.rodkullan.se).

**Breed history from descriptions by breed associations and ([www.fjallko.se](http://www.fjallko.se); rodkullan.se; [www.allmogekon.se](http://www.allmogekon.se); srb-foreningen.se) and Johansson, I. 1941. Svenskt Lantbrukslexikon, p717 (http://runeberg.org/lantblex/0721.html).

# Note that Swedish abbreviation for the breeds are not used in the study.

**Table S2 Information of samples included from the previous studies**

| **Breed** | **Sample Size** | **Original study** | **Reference** |
| --- | --- | --- | --- |
| Limousin | 25 | Matukumalli et al., (2009) | [36] |
| Red Angus | 19 | Decker et al., (2014) | [38] |
| Holstein-Friesian | 25 | Matukumalli et al., (2009) | [36] |
| Gir | 24 | Bahbahani et al., (2017) | [35] |
| Highland | 11 | Decker et al., (2014), Upadhyay et al., (2016) | [38, 10] |
| Norwegian Red | 21 | Matukumalli et al., (2009) | [36] |
| Normande | 25 | Gautier et al., (2009) | [37] |
| Guernsey | 21 | Matukumalli et al., (2009) | [36] |
| Jersey | 25 | Matukumalli et al., (2009) | [36] |
| Galloway | 5 | Upadhyay et al., (2016) | [10] |
| Kerry | 4 | Upadhyay et al., (2016) | [10] |
| Dutch Friesian | 5 | Upadhyay et al., (2016) | [10] |

**Table S3 Cumulative ROH count, ROH length (kb), and average ROH size (kb) per individual**

| **Breed** | **Ind_id** | **ROH_count** | **ROH_length** | **KBAVG** |
| --- | --- | --- | --- | --- |
| Bohus Polled | BHP1 | 31 | 277650 | 8956.44 |
| Bohus Polled | BHP2 | 27 | 176007 | 6518.78 |
| Bohus Polled | BHP3 | 21 | 144995 | 6904.53 |
| Bohus Polled | BHP4 | 41 | 657225 | 16029.9 |
| Bohus Polled | BHP5 | 37 | 391302 | 10575.7 |
| Bohus Polled | BHP6 | 51 | 682650 | 13385.3 |
| Fjällnära | FNC1 | 64 | 1.13E+06 | 17598.9 |
| Fjällnära | FNC2 | 50 | 565256 | 11305.1 |
| Fjällnära | FNC3 | 37 | 575020 | 15541.1 |
| Fjällnära | FNC4 | 43 | 488360 | 11357.2 |
| Fjällnära | FNC5 | 59 | 816211 | 13834.1 |
| Fjällnära | FNC6 | 34 | 396645 | 11666 |
| Fjällnära | FNC7 | 19 | 298669 | 15719.4 |
| Fjällnära | FNC8 | 8 | 60714.8 | 7589.35 |
| Fjällnära | FNC9 | 24 | 289543 | 12064.3 |
| Fjällnära | FNC10 | 42 | 506548 | 12060.7 |
| Fjällnära | FNC11 | 26 | 197392 | 7592.01 |
| Fjällnära | FNC12 | 39 | 515562 | 13219.5 |
| Fjällnära | FNC13 | 37 | 362262 | 9790.87 |
| Fjällnära | FNC14 | 67 | 902550 | 13470.9 |
| Fjällnära | FNC15 | 78 | 1.34E+06 | 17137.8 |
| Fjällnära | FNC16 | 67 | 712271 | 10630.9 |
| Ringamåla | RMC1 | 50 | 322736 | 6454.73 |
| Ringamåla | RMC2 | 28 | 339014 | 12107.6 |
| Ringamåla | RMC3 | 43 | 363115 | 8444.54 |
| Ringamåla | RMC4 | 53 | 490782 | 9260.04 |
| Ringamåla | RMC5 | 41 | 408319 | 9959 |
| Ringamåla | RMC6 | 69 | 777420 | 11267 |
| Ringamåla | RMC7 | 44 | 305851 | 6951.15 |
| Ringamåla | RMC8 | 50 | 402889 | 8057.77 |
| Ringamåla | RMC9 | 54 | 415506 | 7694.55 |
| Ringamåla | RMC10 | 52 | 342095 | 6578.75 |
| Ringamåla | RMC11 | 61 | 480171 | 7871.66 |
| Ringamåla | RMC12 | 57 | 343834 | 6032.18 |
| Ringamåla | RMC13 | 42 | 495563 | 11799.1 |
| Swedish Holstein-Friesian | SHF1 | 19 | 115913 | 6100.67 |
| Swedish Holstein-Friesian | SHF2 | 5 | 25590.7 | 5118.13 |
| Swedish Holstein-Friesian | SHF3 | 20 | 136116 | 6805.78 |
| Swedish Holstein-Friesian | SHF4 | 31 | 295400 | 9529.02 |
| Swedish Holstein-Friesian | SHF5 | 2 | 6622.67 | 3311.34 |
| Swedish Holstein-Friesian | SHF6 | 19 | 144665 | 7613.96 |
| Swedish Holstein-Friesian | SHF7 | 20 | 120126 | 6006.3 |
| Swedish Holstein-Friesian | SHF8 | 11 | 49244.8 | 4476.8 |
| Swedish Holstein-Friesian | SHF9 | 12 | 108631 | 9052.56 |
| Swedish Holstein-Friesian | SHF10 | 26 | 203118 | 7812.24 |
| Swedish Holstein-Friesian | SHF11 | 16 | 104480 | 6529.99 |
| Swedish Holstein-Friesian | SHF12 | 13 | 52090.3 | 4006.94 |
| Swedish Holstein-Friesian | SHF13 | 12 | 55098.7 | 4591.56 |
| Swedish Holstein-Friesian | SHF14 | 27 | 196368 | 7272.88 |
| Swedish Holstein-Friesian | SHF15 | 15 | 73161.4 | 4877.42 |
| Swedish Holstein-Friesian | SHF16 | 14 | 83295.9 | 5949.71 |
| Swedish Holstein-Friesian | SHF17 | 29 | 188446 | 6498.14 |
| Swedish Holstein-Friesian | SHF18 | 15 | 78462.6 | 5230.84 |
| Swedish Holstein-Friesian | SHF19 | 14 | 126180 | 9012.87 |
| Swedish Holstein-Friesian | SHF20 | 20 | 130800 | 6540 |
| Swedish Holstein-Friesian | SHF21 | 8 | 54928.7 | 6866.09 |
| Swedish Holstein-Friesian | SHF22 | 4 | 21910.5 | 5477.63 |
| Swedish Holstein-Friesian | SHF23 | 7 | 33008.1 | 4715.45 |
| Swedish Holstein-Friesian | SHF24 | 18 | 113451 | 6302.81 |
| Fjäll | SMC1 | 1 | 4513.4 | 4513.4 |
| Fjäll | SMC2 | 13 | 105056 | 8081.22 |
| Fjäll | SMC3 | 33 | 233180 | 7066.05 |
| Fjäll | SMC4 | 22 | 142717 | 6487.15 |
| Fjäll | SMC5 | 24 | 145324 | 6055.16 |
| Fjäll | SMC6 | 9 | 81616.3 | 9068.47 |
| Fjäll | SMC7 | 22 | 132280 | 6012.74 |
| Fjäll | SMC8 | 23 | 120029 | 5218.67 |
| Fjäll | SMC9 | 30 | 209008 | 6966.94 |
| Fjäll | SMC10 | 35 | 273677 | 7819.33 |
| Fjäll | SMC11 | 26 | 185434 | 7132.07 |
| Fjäll | SMC12 | 14 | 90293.8 | 6449.55 |
| Fjäll | SMC13 | 19 | 140537 | 7396.69 |
| Fjäll | SMC14 | 25 | 162428 | 6497.13 |
| Fjäll | SMC15 | 13 | 95844 | 7372.61 |
| Fjäll | SMC16 | 3 | 16922.5 | 5640.82 |
| Fjäll | SMC17 | 32 | 230407 | 7200.22 |
| Fjäll | SMC18 | 23 | 125903 | 5474.05 |
| Fjäll | SMC19 | 14 | 118496 | 8464.01 |
| Fjäll | SMC20 | 19 | 156027 | 8211.95 |
| Fjäll | SMC21 | 32 | 245316 | 7666.11 |
| Fjäll | SMC22 | 45 | 310073 | 6890.51 |
| Fjäll | SMC23 | 17 | 69236.8 | 4072.75 |
| Swedish Polled | SPC1 | 22 | 156949 | 7134.06 |
| Swedish Polled | SPC2 | 19 | 183609 | 9663.64 |
| Swedish Polled | SPC3 | 15 | 85702.7 | 5713.52 |
| Swedish Polled | SPC4 | 16 | 152165 | 9510.29 |
| Swedish Polled | SPC5 | 15 | 89125.2 | 5941.68 |
| Swedish Polled | SPC6 | 8 | 69881.6 | 8735.2 |
| Swedish Polled | SPC7 | 2 | 6727.07 | 3363.53 |
| Swedish Polled | SPC8 | 19 | 107293 | 5647 |
| Swedish Polled | SPC9 | 9 | 56020.4 | 6224.49 |
| Swedish Polled | SPC10 | 25 | 140911 | 5636.45 |
| Swedish Polled | SPC11 | 8 | 81398.3 | 10174.8 |
| Swedish Polled | SPC12 | 13 | 85132.1 | 6548.62 |
| Swedish Red | SRC1 | 36 | 279173 | 7754.8 |
| Swedish Red | SRC2 | 27 | 149081 | 5521.53 |
| Swedish Red | SRC3 | 17 | 64561.5 | 3797.74 |
| Swedish Red | SRC4 | 43 | 246863 | 5741 |
| Swedish Red | SRC5 | 35 | 193083 | 5516.65 |
| Swedish Red | SRC6 | 45 | 310187 | 6893.05 |
| Swedish Red | SRC7 | 39 | 257462 | 6601.6 |
| Swedish Red | SRC8 | 50 | 364419 | 7288.39 |
| Swedish Red | SRC9 | 10 | 32527.3 | 3252.73 |
| Swedish Red | SRC10 | 5 | 22134.2 | 4426.84 |
| Swedish Red | SRC11 | 48 | 303545 | 6323.86 |
| Swedish Red | SRC12 | 48 | 458753 | 9557.36 |
| Swedish Red | SRC13 | 48 | 381931 | 7956.9 |
| Swedish Red | SRC14 | 42 | 309950 | 7379.76 |
| Swedish Red | SRC15 | 9 | 39200.5 | 4355.61 |
| Swedish Red | SRC16 | 51 | 273527 | 5363.28 |
| Swedish Red | SRC17 | 23 | 160032 | 6957.92 |
| Swedish Red | SRC18 | 28 | 142384 | 5085.16 |
| Swedish Red | SRC19 | 39 | 236181 | 6055.94 |
| Swedish Red | SRC20 | 16 | 56245 | 3515.31 |
| Swedish Red | SRC21 | 39 | 251070 | 6437.7 |
| Swedish Red | SRC22 | 46 | 314334 | 6833.36 |
| Swedish Red | SRC23 | 7 | 24010 | 3430 |
| Swedish Red Polled | SRP1 | 8 | 43389 | 5423.63 |
| Swedish Red Polled | SRP2 | 10 | 57861.9 | 5786.19 |
| Swedish Red Polled | SRP3 | 22 | 190738 | 8669.9 |
| Swedish Red Polled | SRP4 | 18 | 127012 | 7056.22 |
| Swedish Red Polled | SRP5 | 36 | 523822 | 14550.6 |
| Swedish Red Polled | SRP6 | 74 | 1.36E+06 | 18439.1 |
| Swedish Red Polled | SRP7 | 4 | 22048 | 5512.01 |
| Swedish Red Polled | SRP8 | 35 | 534435 | 15269.6 |
| Swedish Red Polled | SRP9 | 1 | 4349.89 | 4349.89 |
| Swedish Red Polled | SRP10 | 21 | 279306 | 13300.3 |
| Swedish Red Polled | SRP11 | 22 | 318630 | 14483.2 |
| Swedish Red Polled | SRP12 | 20 | 146949 | 7347.45 |
| Swedish Red Polled | SRP13 | 7 | 36785.5 | 5255.07 |
| Swedish Red Polled | SRP14 | 13 | 94936.9 | 7302.84 |
| Swedish Red Polled | SRP15 | 23 | 236040 | 10262.6 |
| Swedish Red Polled | SRP16 | 26 | 244154 | 9390.54 |
| Swedish Red Polled | SRP17 | 8 | 72035 | 9004.37 |
| Väne | VAC1 | 37 | 274061 | 7407.04 |
| Väne | VAC2 | 44 | 353485 | 8033.75 |
| Väne | VAC3 | 43 | 475528 | 11058.8 |
| Väne | VAC4 | 49 | 389932 | 7957.8 |
| Väne | VAC5 | 47 | 256218 | 5451.44 |
| Väne | VAC6 | 45 | 400359 | 8896.86 |
| Väne | VAC7 | 43 | 553236 | 12866 |
| Väne | VAC8 | 36 | 382582 | 10627.3 |
| Väne | VAC9 | 63 | 714836 | 11346.6 |


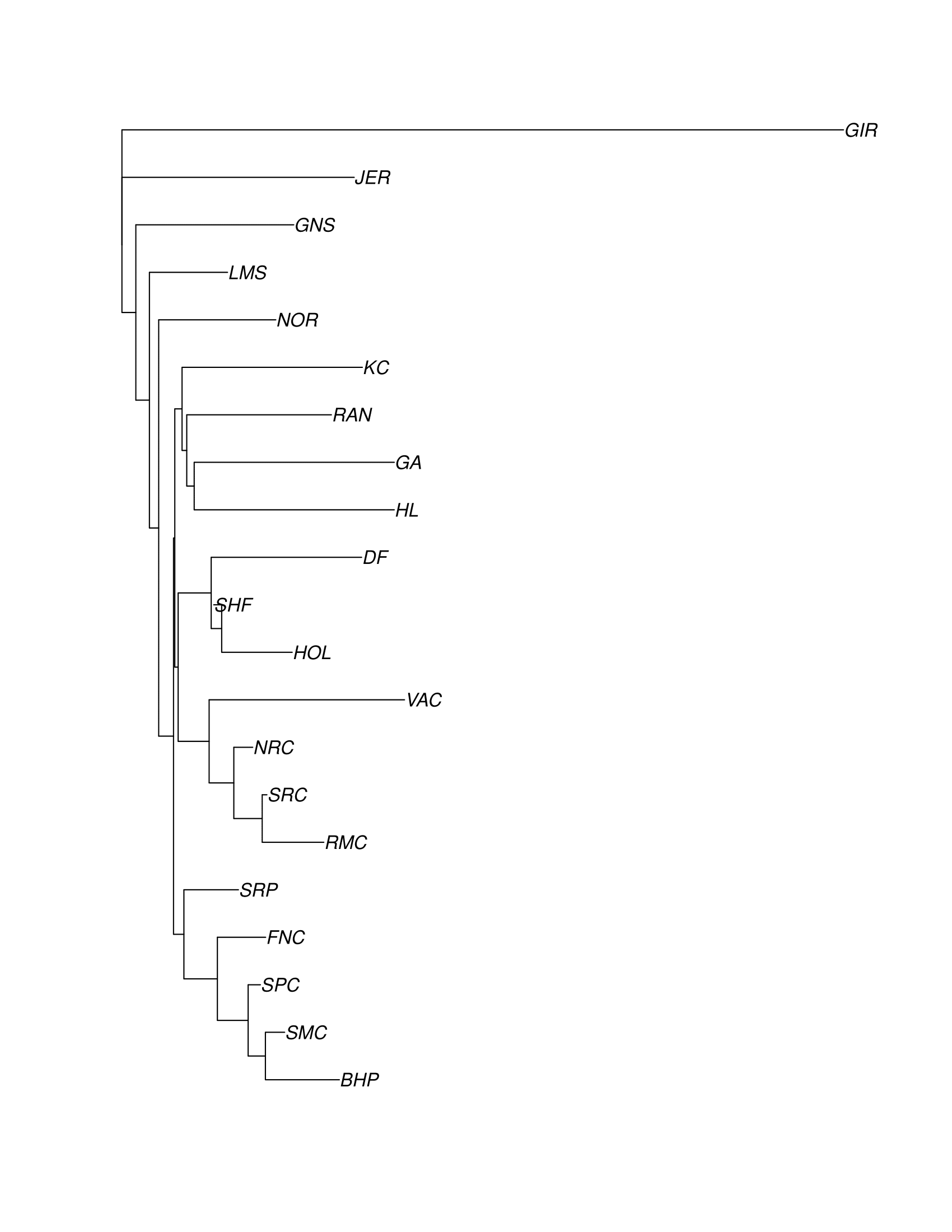


**Figure S1: Fst-based phylogenetic tree showing the relationships between different European cattle breeds.**

Description: The following abbreviations are used: JER-Jersey, GNS: Guernsey, LMS: Limousin, NOR: Normande, KC: Kerry cattle, RAN: Red Angus, GA: Galloway, HL: Scottish Highland cattle, DF: Dutch Friesian, SHF: Swedish Holstein-Friesian, HOL: Holstein-Friesian, VAC: Väne cattle, NRC: Norwegian Red cattle, SRC: Swedish Red cattle, RMC: Ringamåla cattle, SRP: Swedish Red Polled, FNC: Fjällnära cattle, SPC: Swedish Polled cattle, SMC: Swedish Mountain cattle (Fjäll cattle), BHP: Bohus Polled cattle.
