# Supplementary figures and images for: Genomic relatedness and diversity of Swedish native cattle breeds
Source: Genet Sel Evol. 2019 Oct 2;51:56. doi: 10.1186/s12711-019-0496-0 (PMC6775670; doi:10.1186/s12711-019-0496-0)

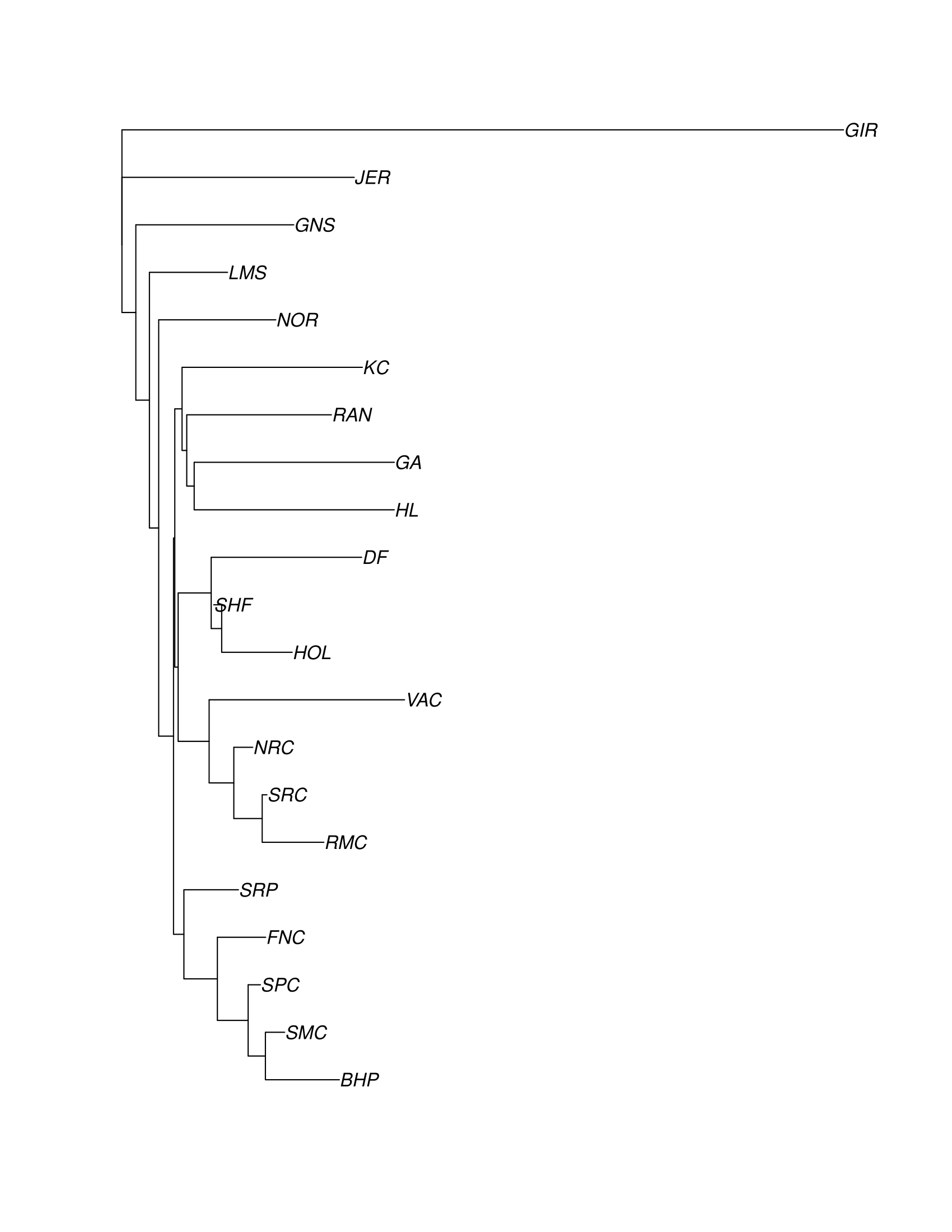

Supplement: Supplementary file 2 — Additional file 2: Figure S1. Fst-based phylogenetic tree showing the relationships between different European cattle breeds. The following abbreviations are used: JER-Jersey, GNS: Guernsey, LMS: Limousin, NOR: Normande, KC: Kerry cattle, RAN: Red Angus, GA: Galloway, HL: Scottish Highland cattle, DF: Dutch Friesian, SHF: Swedish Holstein-Friesian, HOL: Holstein-Friesian, VAC: Väne cattle, NRC: Norwegian Red cattle, SRC: Swedish Red cattle, RMC: Ringamåla cattle, SRP: Swedish Red Polled, FNC: Fjällnära cattle, SPC: Swedish Polled cattle, SMC: Swedish Mountain cattle (Fjäll cattle), BHP: Bohus Polled cattle. [file 12711_2019_496_MOESM2_ESM.png]
